# Progress towards elimination: a case study of Sri Lanka

**DOI:** 10.1186/1475-2875-11-S1-O10

**Published:** 2012-10-15

**Authors:** Gavvrie NL Galappaththv, Cara Smith Gueve, Rabindra R Abeyasinghe, Jim G Kahn, Richard GA Feachem

**Affiliations:** 1 Anti-Malaria Campaign, Ministry of Health, Sri Lanka; 2 Global Health Group, University of California, San Francisco, USA; 3 World Health Organization, Papua New Guinea; 4 Institute for Health Policy Studies, University of California, San Francisco, USA

## 

This abstract is submitted as part of the panel session on case studies for elimination by the WHO Global Malaria Programme and the UCSF Global Health Group.

## Background

As malaria transmission declines and malaria programs shift their focus from malaria control to elimination, it is vital to have documentation of the strategies that countries have used and are currently applying as they seek to eliminate malaria. Our case study of Sri Lanka, which has a long history of malaria control, including a period of near elimination and resurgence in the 1960s, aims to capture the key factors behind the country’s decline in malaria over the last decade.

## Materials and methods

This case study employed qualitative and quantitative methods, using data triangulation to compare and contrast trends. A review of available literature was conducted, and district and national data were collected on incidence, surveillance and vector control. Trends were observed across years and districts, in particular comparing conflict and non-conflict districts. Thirty-three key informant interviews were conducted. Expenditures in two districts for two years were compiled to identify changes in expenditure.

## Results

Malaria control in Sri Lanka began in the early 1900s [1]. Indoor residual spraying (IRS) was introduced in 1945 and, in combination with surveillance, led to a decline in cases to 17 in 1963 [1]. Only four years later, however, two outbreaks of *P. vivax* in 1967 led to a major epidemic (1967-68) [2]. Factors contributing to the epidemic were the reduction of DDT, emerging vector resistance to DDT, complacency of malaria control officers, lack of funding and population movement [2-4] As a result Sri Lanka again scaled up IRS mobile units but the damage had been done. In the last decade, Sri Lanka has made great strides in reducing its malaria burden. Malaria incidence in Sri Lanka has declined by 99.9% since 1999. During this time, there were major increases in the proportion of malaria infections due to *Plasmodium vivax*, and those occurring in adult males. New vector control strategies were introduced, such as spatial insecticide rotation and long-lasting insecticide-treated nets. A strong passive case detection system is the foundation for diagnosis, while active case detection grew from identifying 1.1% of all infections in 2000 to 13.1% in 2007. Vector control and surveillance measures were maintained in conflict areas. For example, coverage of indoor residual spraying of risk populations in conflict districts was 45.9% in 2005 (10.9% in non-conflict districts). One of two districts in the study reported a 48% decline in malaria programme expenditure per person at risk from 2004 to 2009, and a decline in prevention costs and an increase in surveillance costs.

## Conclusions

Malaria is now at low levels in Sri Lanka - 124 indigenous cases were found in 2011. Evidence-driven policy and an ability to adapt to new challenges contributed to this decline.
